# Effectiveness of Selective Laser Trabeculoplasty Applied to 360° vs. 180° of the Angle

**DOI:** 10.1155/2021/8860601

**Published:** 2021-02-16

**Authors:** Abraham Nirappel, Emma Klug, Rebecca Ye, Nathan Hall, Marika Chachanidze, Ta C. Chang, David Solá-Del Valle

**Affiliations:** ^1^Case Western Reserve University School of Medicine, Cleveland, OH, USA; ^2^Massachusetts Eye and Ear Infirmary, Department of Ophthalmology, Harvard Medical School, Boston, MA, USA; ^3^Bascom Palmer Eye Institute, University of Miami Leonard M. Miller School of Medicine, Miami, FL, USA

## Abstract

**Purpose:**

To compare the effectiveness and safety of 360° and 180° of Selective Laser Trabeculoplasty (SLT) for the treatment of elevated intraocular pressure (IOP).

**Methods:**

Retrospective cohort study. The main outcome measure was the Kaplan–Meier analysis comparing the cumulative probabilities of survival between the 360° and 180° SLT groups in terms of IOP reduction. Success was defined as ≥20% IOP reduction from baseline with an IOP between 5–18 mmHg and ≤1 glaucoma medication added postoperatively. Additional outcome measures included changes in average IOP, number of glaucoma medications, and the incidence of postoperative IOP spikes. Measurements were obtained at 6 weeks, 1 year, and 2 years postoperatively.

**Results:**

Two hundred and fifty-eight eyes of 258 patients were included in the 360° group, and 196 eyes of 196 patients were included in the 180° group. The mean IOP reductions at 2 years were 2.21 ± 2.02 mmHg and 2.43 ± 1.81 mmHg (*p*=0.33) in the 180° and 360° groups, respectively. There were no significant differences in the incidence of postoperative IOP spikes between the two groups. There was a significant difference in the survival curves of the two groups (*p*=0.035). The Cox proportional-hazard model indicated that 360° of SLT application was a significant predictor of long-term success (*p*=0.030).

**Conclusions:**

360° of SLT application seems to provide for greater long-term IOP control than 180° of application without putting patients at an elevated risk for postoperative IOP spikes.

## 1. Introduction

Selective Laser Trabeculoplasty (SLT) (Lumenis, Coherent, Inc, Palo Alto, CA, USA) is the most commonly performed procedure to lower intraocular pressure (IOP) in open-angle glaucoma patients [1]. It is generally regarded as effective and safe. Prior studies have indicated that the IOP-reducing ability of SLT is comparable to that of topical glaucoma drops [2–4]. However, SLT may be preferred over topical drops as it potentially avoids any associated side effects as well as issues that may arise from variable patient adherence [1, 5].

It has been well established that the effects of SLT are not permanent. Specifically, prior studies have indicated that approximately 50% of SLT treatments fail within a 2-year period and require further treatment [1, 2]. However, the comparative longevity of 180° and 360° of SLT application has not been definitively established. Previous studies have reported conflicting results regarding the IOP-reducing ability of applying the laser to 180° of the angle versus all 360° [6–9] (Table 1). While some have indicated that the IOP-reducing ability of 360° of SLT application is superior to that of 180° of application, others have shown no such difference.

Additionally, there is still uncertainty about the relative safety of applying the laser to 360° versus 180° of the angle. While SLT has been established as a safe procedure, there is some concern about the risk of both immediate and long-term IOP spikes [1, 10]. To reduce the likelihood of an IOP spike, some practitioners may opt to treat 180° or 90° of the angle instead of the full 360° [7, 8, 11]. There is also uncertainty surrounding the influence of the total laser energy used and the amount of pigmentation in the trabecular meshwork on the likelihood of inducing IOP spikes [12, 13].

The purpose of this study is to compare 360° and 180° of SLT in terms of IOP reduction and safety. To our knowledge, this is the largest study that compares primary 180° of SLT to primary 360° of SLT, with among the longest follow-up periods. Prior studies that have compared 180 and 360° of SLT have typically had fewer patients with follow-up periods of less than two years. In addition, this study evaluates the safety of 180° versus 360° of SLT, specifically in regard to immediate post-laser IOP spikes. Such a comparison has not yet been made in the literature. It also examines whether other covariates, such as baseline IOP, glaucoma severity, glaucoma type, and number of topical glaucoma medications used, have any bearing on the effectiveness of SLT.

## 2. Methods

### 2.1. Study Design

Following approval by the Partners Healthcare Institutional Review Board, we performed a retrospective case-control study of patients who received SLT at Massachusetts Eye and Ear (MEE) from March 2013 to March 2018. Patients were identified using financial claims data (Current Procedural Terminology codes 65855), and all identified patient records were reviewed. Patient who were treated bilaterally had the first treated eye included in the analysis. Data collection methods abided by the Declaration of Helsinki and the Health Portability and Accountability Act.

Patients were included in the analysis if they had at least 6 weeks of follow-up and were at least 18 years of age at the time of the procedure. Patients were excluded if they had any history of SLT in the past.

The following preoperative baseline data were included: age, sex, IOP, lens status, amount of pigmentation present in the trabecular meshwork (as assessed by clinician on gonioscopy), glaucoma type and stage, prior glaucoma surgery, and number of glaucoma medications. Baseline IOP was calculated as an average of the IOP readings from two visits immediately preceding treatment. Preoperative IOP readings and IOP readings at follow-up visits were taken using the Goldmann applanation tonometer. The IOP readings taken on the day of the procedure to reveal immediate IOP spikes were taken with the Tonopen (Reichert Technologies, Depew, NY). Glaucoma severity was determined as mild, moderate, or severe as previously described or as indeterminate if automated visual field data were not available [14]. Fixed-dose combination glaucoma medications were counted by the number of their constituent agents.

Intraoperative data collected included laser power used, duration of application, and degrees of treatment. Mean energy delivered during treatment was calculated by multiplying the power and total duration of treatment as stated in the operative note. Postoperative data were collected at postoperative week 6, year 1, and year 2. Postoperative data on IOP, number of glaucoma medications, and subsequent IOP-lowering procedures were recorded [15].

### 2.2. Outcome Measures

The primary outcome measure was the Kaplan–Meier (KM) survival comparing the cumulative probabilities of success between the 180° and 360° groups. Success was defined as ≥20% IOP reduction from baseline with an IOP between 5–18 mmHg and ≤1 glaucoma medication added postoperatively. Failure was defined as an inability to meet the success criteria for two consecutive follow-up visits, with the latter follow-up visit being used as the failure date. Patients who received any additional IOP-lowering procedure were counted as failures on the date of the additional procedure.

Additional outcome measures included comparisons of the average reductions in IOP, glaucoma medication burden, and the proportions of patients who achieved ≥20% reductions in IOP.

### 2.3. Statistical Analysis

A KM curve was generated to display cumulative survival probabilities. A log-rank test was conducted to test for significant differences in the survival probabilities between the 180° and 360° groups. A Cox proportional-hazard regression analysis was conducted to determine the effects of any baseline characteristics on the hazard of failure.

Chi-squared tests were used to determine any between-group differences in the proportion of patients who achieved at least a 20% reduction in IOP from the preoperative visit at any of the follow-up visits. Chi-squared tests were also used to check if the two groups differed significantly in terms of any baseline characteristics and to compare the incidence of immediate IOP spikes between the two groups. Postoperative IOP spikes of ≥5 mmHg, ≥10 mmHg, ≥30 mmHg, or ≥20% from baseline were each evaluated separately. Pearson correlation coefficients were used to elucidate any statistically significant correlations between the pigmentation, total energy level of the laser, and the incidence of IOP spikes. Patients who required any additional IOP-lowering procedure following SLT, such as trabeculectomy, Micropulse Transscleral Cyclophotocoagulation, repeat SLT, or glaucoma-valve insertion were categorized as failures for the remainder of their follow-up visits.

Two sample *t*-tests were used to determine if there were any significant differences from baseline between the two groups in terms of mean IOP reduction and changes in glaucoma medication burden. All statistical tests were performed at a 5% significance level. All statistical analysis was performed using R (Version 3.6.2).

## 3. Results

### 3.1. Demographics

Data were obtained from 454 eyes of 454 patients who received primary SLT between March 2013 and March 2018 at MEE. Due to patients either being lost to follow-up or having their follow-ups scheduled for a future date, the sample size at each follow-up visit was smaller than that of the original cohort. A total of 438 patients were included in the analysis at the 6-week follow-up visit, 355 were included for the 1-year visit, and 162 for the 2-year visit. Certain patients were not included in the analysis for the 6-week follow-up visit but were included for later visits. Demographic and baseline characteristics are summarized in Table 2. The 180° group had a significantly higher mean baseline IOP and number of glaucoma medications, as well as a significantly higher proportion of patients with severe glaucoma. The 360° had a significantly higher proportion of patients with normal tension and mild glaucoma.

### 3.2. Effectiveness

**Figure 1:**
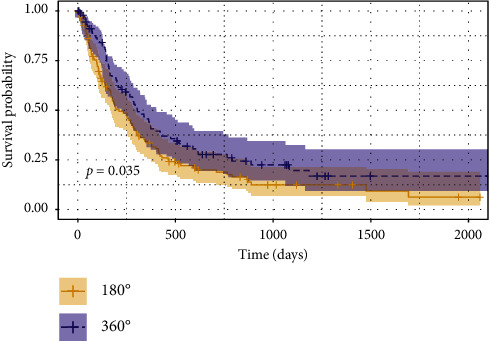
Kaplan–Meier curve comparing the cumulative probabilities survival following 180° and 360° of SLT (Lumenis, Coherent, Inc, Palo Alto, CA, USA). The shaded areas around each plot represent the 95% confidence bands. Success was defined as ≥20% IOP reduction from baseline with an IOP between 5–18 mmHg and ≤1 glaucoma medication added postoperatively. A failure was recorded if a patient either failed to meet success criteria at two consecutive follow-up visits or required an additional IOP-lowering procedure. The log-rank test was used to detect statistical differences between the curves. IOP = intraocular pressure; SLT = Selective Laser Trabeculoplasty; mmHg = millimeters of mercury.

The KM survival curve is displayed in Figure 1. There was a significant difference in the survival probability in terms of IOP reduction between 180° and 360° of SLT (*p*=0.035, log-rank test).

Holding all else constant, the probability of achieving success at any time point with 360° of SLT was 1.40 times more likely than with 180° of SLT (*p*=0.030). Higher baseline IOP was found to be a statistically significant predictor of success, with each one unit increase in baseline IOP reducing the hazard of clinical failure by 4% (*p*=0.022). None of the hazard ratios for any of the other baseline characteristics (age, sex, number of preoperative medications, type of glaucoma, and severity of glaucoma) from the Cox proportional-hazard regression analyses had a statistically significant effect on success rates.

**Figure 2:**
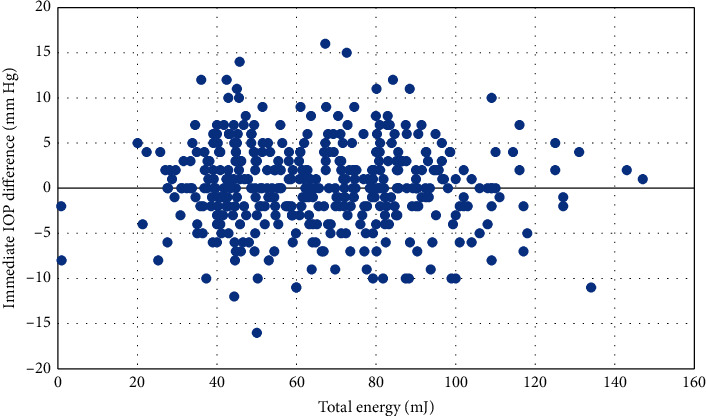
Difference in IOP immediately following SLT and the total energy level of the laser. There was no correlation between the total energy level of the laser and the immediate IOP difference (*r* = −0.01) CI [−0.085, 0.105] (*p*=0.837). IOP = intraocular pressure; SLT = Selective Laser Trabeculoplasty; *r* = Pearson correlation coefficient; CI = confidence interval.

The proportion of patients who had ≥20% reduction in IOP following SLT was greater in the 360° group at each follow-up visit; however, this difference did not become statistically significant until the 2-year follow-up visit (Table 3). While the 360° had a higher average IOP reduction at each follow-up visit, this difference never achieved statistical significance (Table 3). There was no correlation between the total energy level of the laser and the incidence of IOP spikes (*r* = −0.01) CI [−0.085, 0.105] (*p*=0.837) (Figure 2). There was also no correlation between the pigmentation of the trabecular meshwork and the incidence of IOP spikes (*r* = −0.044) CI [−0.190, 0.105] (*p*=0.565). There were no significant differences in the reduction in glaucoma medication burden at any of the follow-up visits (Table 3).

### 3.3. Safety

There was no significant difference in the incidence of immediate IOP spikes of ≥5 mmHg, ≥10 mmHg, or ≥20% from baseline between the two groups. There were no IOP spikes of ≥30 mmHg in either group. There was also no significant difference in the mean duration of time between the laser administration and postoperative IOP check between the two groups (Table 4).

## 4. Discussion

The results of this study suggest that 360° of SLT may confer greater long-term IOP reduction than 180° of SLT without compromising safety. The results of the KM analysis along with the chi-squared tests indicate that the difference in the IOP-reducing ability of 360° and 180° of SLT application becomes more pronounced over time. Past studies comparing 90° or 180° vs. 360° of SLT have not consistently demonstrated that treating greater portions of the trabecular meshwork is associated with greater IOP reduction. In a prospective study where a group of 40 patients were randomized to receive either 180° or 360° of SLT, Goyal et al. demonstrated no significant difference in the IOP reduction between the two groups at the 1-month follow-up visit [9]. Additionally, in a prospective study of 52 eyes by Ozen et al., 26 patients were given both 180° and 360° of SLT treatment. Here, 180° of SLT was applied to one of the patient's eyes, while 360° of SLT was applied to the other. This study found no significant difference in the proportion of eyes which achieved ≥20% reduction in IOP at any point in the 6-month follow-up period. At 6 months, 73.1% of eyes in the 180° group and 76.9% of eyes in the 360° group had maintained ≥20% reduction in IOP [6].

In contrast, other studies have indicated that the IOP-reducing ability of 360° of SLT is demonstrably superior to that of 180° of application. Specifically, in a prospective study of 34 total eyes with a mean follow-up period of 19.5 months, Shibata et al. found a significantly higher average IOP reduction following 360° of application [7]. At the 6-month follow-up visit, the average IOP reduction was 5.6 mm Hg in the 360° group and 2.6 mm Hg in the 180° group (*p* < 0.05). The Kaplan–Meier analysis showed that the long-term success rate, defined as maintaining ≥20% reduction in IOP, was significantly higher in the group which received 360° of treatment [7]. In a prospective trial of 167 eyes, Nagar et al. also demonstrated that 360° of SLT application seemed to be more effective at lowering IOP than 180° of application. While the differences in the proportion of patients who achieved proportion of patients who achieved ≥20% reduction in IOP did not reach significance in the 12-month follow-up period used, it was notably higher in the 360° group than in the 180° group (82% vs. 65%) [8].

In the present study, the proportion of patients who achieved ≥20% reduction in IOP at 2 years was 48% and 29% in the 360° and 180° groups, respectively. This was notably less than the reduction observed in past studies of SLT. As has been established in the literature, preoperative IOP is the greatest predictor for the effectiveness of SLT. With a baseline IOP of 19.3 mm Hg in the 180° group and 18.2 mm Hg in the 360° group, the mean baseline IOPs seen in this study were the lowest out of all the studies examined, which could account for the seemingly smaller IOP-reducing effect of SLT observed here. The Cox Proportional-Hazard model indicates that the differences in IOP reduction between 180° and 360° of SLT maintain statistical significance when the baseline IOPs are held constant. Additionally, it demonstrates that no other pretreatment characteristics, such as type or stage of glaucoma, had a significant effect on the amount of IOP reduction observed. It is also important to note that MEEI serves as a tertiary referral center and the patients included in this study are expected to have more severe glaucoma than average. Some of the previously published work excluded patients with advanced visual field defects, systemic steroid use, and previous eye surgeries. [2, 3, 8] No such exclusions were made in this study, which could contribute to the observed disparity in the success rates of SLT.

One potential concern with applying SLT to all 360° as opposed to 180° of the angle is the potentially greater risk of inducing postoperative complications, such as immediate IOP spikes [1, 10]. This hypothesis was not supported in our study, as there was no significant difference in the incidence of immediate IOP spikes between the two groups. Interestingly, the group which received 180° of SLT treatment in our study was more likely to experience SLT spikes of ≥5 mm Hg, ≥10 mm Hg, or ≥20% from baseline, though these differences did not reach statistical significance. Although there were not any studies in the literature which directly compared the incidence of IOP spikes after 180° and 360° of treatment, Wong et al. found that there were no significant differences in the likelihood of inducing IOP spikes between patients treated with 120 and 160 spots of SLT treatment at 1 hour following treatment [10]. In another study comparing the intervisit IOP fluctuations following 180° and 360° of treatment, Prasad et al. showed that 180° of SLT treatment was associated with significantly greater IOP fluctuations than 360° of treatment at each follow-up visit [16]. While Prasad et al. did not compare the incidence of immediate IOP spikes induced by 180° and 360° of SLT treatment immediately following the laser treatment as we did, the underlying mechanism leading to the significantly higher IOP fluctuations following 180° of SLT treatment may help explain why the 180° group in the present study seemed to have a higher incidence of IOP spikes. One explanation they proposed is that although the 180° of SLT treatment was able to provide sufficient aqueous outflow to reduce IOP, it may not provide enough improvement in outflow during the peaks of IOP, leading to the greater amounts of fluctuation [16]. The greater efficacy of the 360° SLT treatment in reducing the IOP fluctuation when compared with 180° SLT treatment may also be explained by the segmental aqueous outflow through Schlemm's canal, as it has been suggested that the segmental division of Schlemm's canal might be more marked in eyes with glaucoma than in normal eyes [16].

This study includes data up to the 2-year follow-up visit of patients following SLT, which is among the longest follow-up periods in studies examining SLT. While only 162 patients were followed up for the full 2 years, this cohort is still amongst the largest groups studied to date. The generalizability of this study is limited by its retrospective design. Lastly, as the study cohorts are managed at a tertiary referral center, we cannot exclude a systematic referral bias, which may make the findings less generalizable to non-tertiary providers. In summary, these results indicate that 360° of SLT application may be more effective than 180° of SLT application at controlling long-term IOP without sacrificing safety. Future research in the form of a large prospective clinical trial is warranted to determine if these findings hold true over a longer time period.

## Figures and Tables

**Table 1:** Comparison of the main outcomes of past studies comparing 180° and 360° of SLT.

Author, year	Shibata et al. 2012	Ozen et al. 2020	Goyal et al. 2010	Nagar et al. 2005
Type of study	RCS	RCT	RCT	RCT
180°				
F/U (mths)	36	6	1	12
# of eyes	35	26	19	49
Mean preop. IOP (mmHg)	19.5	27.4	26	n/a
Mean reduction (mmHg)	2.6	9.1	6.1	n/a
% IOP reduction	13.3	33.2	23.6	n/a
Proportion with 20% IOP reduction	n/a	73.1	72.1	65
360°				
F/U (mths)	36	6	1	12
# of eyes	34	26	18	44
Mean preop. IOP (mmHg)	21.0	27.7	25.5	n/a
Mean reduction (mmHg)	5.6	10.3	5.7	n/a
% IOP reduction	26.7	37.2	22.4	n/a
Proportion with 20% IOP reduction	n/a	76.9	89.5	82

F/U = maximum follow-up period; RCS = retrospective cohort study; RCT = randomized clinical trial; IOP = intraocular pressure; mmHg = millimeters of mercury.

**Table 2:** Baseline characteristics.

Baseline characteristics	180°	360°	*p* value^*∗*^
IOP (mm Hg ± SD)	19.3 ± 3.8	18.2 ± 4.4	0.01^*∗*^
Number of eyes	196	258	
Mean age (±SD)	67.8 ± 11.7	67.6 ± 13.3	0.92
Age (range)	18–97	21–94	
Sex (% female)	53	52	0.87
Medications (±SD)	2.67 ± 1.38	1.98 ± 1.34	<0.01^*∗*^
Pseudophakes (%)	0.68 ± 0.54	0.84 ± 0.98	0.28

Type of glaucoma, *N* (%)			*p* value
Ocular hypertension	20 (10)	23 (9)	0.78
POAG	128 (65)	191 (74)	0.05
PXFG	22 (11)	13 (5)	0.07
Pigmentary	8 (4)	3 (1)	0.14
NTG	6 (3)	18 (7)	0.03^*∗*^
MMG	10 (5)	10 (4)	0.53
Uveitic	2 (1)	0 (0)	0.11

Severity of glaucoma, *N* (%)			*p* value
Ocular hypertension	31 (16)	29 (11)	0.12
Mild	39 (20)	80 (31)	<0.01^*∗*^
Moderate	53 (27)	90 (35)	0.08
Severe	73 (37)	59 (23)	<0.01^*∗*^

IOP = intraocular pressure; SD = standard deviation; mmHg = millimeters of mercury; POAG = primary open-angle glaucoma; PXFG = pseudoexfoliation glaucoma; NTG = normal tension glaucoma; MMG = mixed-mechanism glaucoma.

**Table 3:** Comparison of average IOP reductions between the 180° and 360° SLT groups.

IOP reduction	180°	360°	*p* value
Week 6			
*n*	190	248	
Average IOP reduction (mmHg ± SD)	2.85 ± 1.9	3.16 ± 2.05	0.44
≥20% IOP reduction (%)	40	48	0.134
Mean medication reduction	0.05 ± 0.93	0.06 ± 0.89	0.98

Year 1			
*n*	166	189	
Average IOP reduction (mmHg ± SD)	2.97 ± 2.06	3.40 ± 2.22	0.39
≥20% IOP reduction (%)	34	42	0.248
Mean medication reduction	0.08 ± 0.91	0.11 ± 0.82	0.88

Year 2			
*n*	75	87	
Average IOP reduction (mmHg ± SD)	2.21 ± 2.02	2.43 ± 1.81	0.33
≥20% IOP reduction (%)	29	48	0.01^*∗*^
Mean medication reduction	0.08 ± 0.96	0.14 ± 0.91	0.73

IOP = intraocular pressure; mm Hg = millimeters of mercury; (SD) = standard deviation; *n* *=* number of eyes at follow-up visit.

**Table 4:** Comparison of immediate IOP spikes and mean time to IOP check.

Immediate IOP spikes	180°	360°	*p* value
Mean time to IOP check (min ± SD)	94.2 ± 6.35	91.2 ± 5.95	0.202
IOP spike ≥5 mmHg (%)	21	14	0.09
IOP spike ≥10 mmHg (%)	5.8	3.3	0.26
IOP spike ≥20% from baseline (%)	26.1	19.8	0.17

IOP = intraocular pressure; min = minutes; mmHg = millimeters of mercury; (SD) = standard deviation.

## Data Availability

Data are available upon request; please contact abraham.nirappel@gmail.com for access. De-identified data may be provided according to Partners IRB regulations.
